# MicroRNAs and long non-coding RNAs in cartilage homeostasis and osteoarthritis

**DOI:** 10.3389/fcell.2022.1092776

**Published:** 2022-12-13

**Authors:** Jingliang Gu, Wu Rao, Shaochuan Huo, Tianyou Fan, Minlei Qiu, Haixia Zhu, Deta Chen, Xiaoping Sheng

**Affiliations:** ^1^ Department of Orthopedics, Shanghai Municipal Hospital of Traditional Chinese Medicine, Shanghai University of Traditional Chinese Medicine, Shanghai, China; ^2^ Shenzhen Hospital of Guangzhou University of Chinese Medicine, Shenzhen, China

**Keywords:** osteoarthritis, cartilage homeostasis, microRNA, lncRNA, regeneration

## Abstract

During the last decade, osteoarthritis (OA) has become one of the most prevalent musculoskeletal diseases worldwide. OA is characterized by progressive loss of articular cartilage, abnormal remodeling of subchondral bone, hyperplasia of synovial cells, and growth of osteophytes, which lead to chronic pain and disability. The pathological mechanisms underlying OA initiation and progression are still poorly understood. Non-coding RNAs (ncRNAs) constitute a large portion of the transcriptome that do not encode proteins but function in numerous biological processes. Cumulating evidence has revealed a strong association between the changes in expression levels of ncRNA and the disease progression of OA. Moreover, loss- and gain-of-function studies utilizing transgenic animal models have demonstrated that ncRNAs exert vital functions in regulating cartilage homeostasis, degeneration, and regeneration, and changes in ncRNA expression can promote or decelerate the progression of OA through distinct molecular mechanisms. Recent studies highlighted the potential of ncRNAs to serve as diagnostic biomarkers, prognostic indicators, and therapeutic targets for OA. MiRNAs and lncRNAs are two major classes of ncRNAs that have been the most widely studied in cartilage tissues. In this review, we focused on miRNAs and lncRNAs and provided a comprehensive understanding of their functional roles as well as molecular mechanisms in cartilage homeostasis and OA pathogenesis.

## Introduction

The maintenance of cartilage homeostasis is crucial for our joint function because the loss of cartilage homeostasis can cause extracellular matrix (ECM) degradation and chondrocyte death, leading to irreversible cartilage damage and the onset of osteoarthritis (OA) (Tong et al., 2022). During the last decade, OA has become the most prevalent degenerative joint condition affecting more than 25% of the population over 18 years of age (Chen et al., 2017). Typical pathological changes in an OA joint include gradual loss and degradation of articular cartilage, sclerosis of the subchondral bone, growth of osteophytes, synovial inflammation, ligament, and meniscal lesions, and enlargement of the joint capsule (Chen et al., 2017; Snoeker et al., 2020; Tong et al., 2022). The risk factors for developing OA include genetic susceptibility, aging, obesity, and joint injury (Palazzo et al., 2016). There are currently no effective therapies to repair damaged cartilage or slow the progression of the disease since the precise molecular mechanisms of OA pathogenesis are still poorly understood. Interestingly, cumulating evidence has recently revealed that non-coding RNAs (ncRNAs) play multiple central roles in maintaining cartilage homeostasis and are also deeply involved in OA pathogenesis (Razmara et al., 2019; Xie et al., 2020). The ncRNAs refer to functional RNAs that are transcribed from DNA but not translated into proteins (Mattick and Makunin, 2006). It is well-known that the ncRNAs can function as epigenetic regulators in regulating gene and protein expressions and thus participate in numerous fundamental biological processes (Wei et al., 2017). Epigenetic-related ncRNAs include microRNA (miRNA), small interfering RNA (siRNA), piwi-interacting RNA (piRNA), circular RNA (circRNA), and long non-coding RNA (lncRNA) (Wei et al., 2017). The ncRNAs can act as vital regulators in cartilage formation and homeostasis by mediating a series of physiological processes, such as chondrocyte proliferation, differentiation, and ECM biosynthesis (Gibson and Asahara, 2013; Huang et al., 2019; Razmara et al., 2019). Dysregulation in ncRNAs expression can lead to ECM degradation, chondrocyte hypertrophy, senescence, and apoptosis, ultimately resulting in OA initiation and progression (Weilner et al., 2015; Xie et al., 2020; Pekacova et al., 2022). Recent studies underscored the potential of ncRNAs as diagnostic biomarkers, prognostic indicators, and therapeutic targets for OA (Ghafouri-Fard et al., 2021).

MiRNAs and lncRNAs are two major classes of ncRNAs that have been the most widely studied in the regulatory mechanisms of skeletal homeostasis and diseases. MiRNAs are small non-coding RNAs (∼21–24 nucleotides in length) with essential biological activities, such as post-translational regulation of the gene expression (Saquib et al., 2021). The synthesis of miRNAs begins with the transcription of DNA sequences into primary miRNAs (pri-miRNAs). Then the pri-miRNAs are processed into precursor miRNAs (pre-miRNAs) and eventually mature miRNAs through several sophisticated molecular mechanisms (O'Brien et al., 2018; Adams, 2017; Joyce et al., 2018). LncRNAs, which are RNA transcripts longer than 200 nucleotides, represent a considerable portion of ncRNAs. The biogenesis of lncRNA is highly similar to that of messenger RNAs (mRNAs), a process that includes polymerase II-mediated transcription, polyadenylation, splicing, and 5′-capping (Kung et al., 2013; Aliperti et al., 2021). The processes controlling lncRNA synthesis are cell type- and stage-specific and governed by cell type- and stage-specific stimuli (Dahariya et al., 2019). Once lncRNAs finish their transcription, they fold into thermodynamically stable secondary structures and display distinct expression patterns and specific nuclear localization, which differs from mRNAs (Saxena and Carninci, 2011). In this review, we focused on miRNAs and lncRNAs and provided a comprehensive understanding of their functional roles in cartilage homeostasis and OA pathogenesis.

### MiRNAs maintain cartilage homeostasis

Recent studies have highlighted a series of crucial functions of miRNAs in maintaining cartilage homeostasis (Razmara et al., 2019; Fujii et al., 2022). *In vitro* chondrogenesis of mesenchymal stem cells is the most common experiment used to study the role of miRNAs in chondrocyte proliferation and chondrogenic differentiation. Meng and coworkers showed that miR-193b-3p expression was elevated in chondrogenic and hypertrophic human mesenchymal stem cells (hMSC) and was significantly decreased in degenerated human cartilage. MiR-193b-3p regulates hMSC chondrogenesis and the metabolism of primary human chondrocytes by directly targeting the 3′-untranslated region (3′-UTR) of histone deacetylase 3 (HDAC3) mRNA (Meng et al., 2018). MiR-200a was reported to have a role in controlling the proliferation and differentiation of mandibular condylar chondrocytes (MCCs) (Umeda et al., 2015). Transfection of miR-200a mimics inhibits MCC differentiation and promotes cell proliferation, while miR-200a inhibitors enhance MCC differentiation. MiR-9 regulates the survival of chondroblasts by targeting protogenin and thus maintains the cartilage homeostasis (Song et al., 2013). MiR-142-3p inhibits chondrocyte apoptosis and inflammation through targeting the expression of high mobility group box 1 (HMGB1), an essential pathological factor leading to the loss of cartilage homeostasis (Wang et al., 2016). MiR-140 is specifically expressed in healthy cartilage and has crucial functions in maintaining ECM homeostasis, probably by promoting the production of type II collagen and inhibiting the expressions of matrix metalloproteinase 13 (Mmp13) and a disintegrin and metalloproteinase with thrombospondin motifs 5 (Adamts5) (Si et al., 2017). Using genetically modified mouse models, Huang et al. showed that two homologous miRNAs, miR-204 and miR-211, synergistically maintain cartilage homeostasis and protect cartilage from OA-like lesions (Huang et al., 2019). The absence of miR-204/-211 in mesenchymal progenitor cells (MPCs) causes abnormal accumulation of Runx2, a vital transcriptional factor for promoting chondrocyte hypertrophy in multi-type joint cells, which leads to OA-like degeneration of the whole joint. Moreover, loss of miR-204/-211 expression strongly stimulates matrix-degrading proteases, such as Mmp13 and Adamts5 in articular chondrocytes and synoviocytes and thus promote the degradation of articular cartilage. Furthermore, overexpression of miR-204 in the articular joint essentially restores cartilage homeostasis and decelerates the progression of OA in mice. Both strands of miR-455, i.e., miR-455-5p and miR-455-3p, are highly expressed in chondrocytes of healthy articular cartilage (Ito et al., 2021). Genetic ablation of miR-455 disrupts cartilage homeostasis and results in considerable OA-like damages, whereas overexpression of both strands of miR-455 protects against surgery-induced cartilage degradation in mice (Ito et al., 2021). Zhang et al. recently reported that miR-17 is strongly expressed in chondrocytes of both superficial and middle zone cartilages under homeostatic conditions (Zhang et al., 2022). The expression of miR-17 is essential for maintaining the balance between ECM anabolism and catabolism.

In response to mechanical loading, chondrocyte-mediated mechanotransduction is necessary for cartilage health and homeostasis (Campbell et al., 2007). Recent studies have revealed the participation of miRNAs in mechanotransduction within articular cartilage. By utilizing the miRNA microarray technology, the expression pattern of miRNAs in the anterior weight-bearing zone and posterior non-weight-bearing area of bovine articular cartilage was analyzed (Dunn et al., 2009). The data showed that miR-221 and miR-222 were markedly higher in the anterior weight-bearing medical condylar cartilage compared to the posterior non-weight-bearing medial condylar cartilage, implicating potential roles of these miRNAs in chondrocyte mechanotransduction. Shang and coworkers further studied the extracellular vehicles (EVs)-mediated intercellular communication between chondrocytes and osteoblasts (Shang et al., 2021). They found that chondrocyte-derived EVs contain miR-221-3p, and the latter can be transferred to osteoblasts to regulate gene expressions (Shang et al., 2021). Collectively, these findings suggest that the expressions of specific miRNA clusters are necessary for the maintenance of cartilage homeostasis. Loss of these homeostatic miRNAs may lead to cartilage degeneration and OA.

### MiRNAs mediate cartilage regeneration

Articular cartilage has a limited potential for self-repair. Cartilage regeneration technology has been developed to produce resilient cartilage-like tissue where cartilage has been worn away or destroyed (Xiang et al., 2022). Surgical intervention is often required for cartilage regeneration (Murphy et al., 2020). The most common arthroscopic treatment for cartilage injuries is chondroplasty or removing loose cartilage fragments (Martin et al., 2019). This gives temporary symptomatic alleviation; nevertheless, the remaining cartilage is more prone to wear and rapid degeneration. Microfracture is another frequently used method that pierces the subchondral bone to let bone marrow fill the cartilage defects. This method leads to the production of fibrocartilage tissue that is mechanically inferior. Recent research has underscored miRNAs as a potential therapeutic agent for promoting cartilage regeneration (Foo et al., 2021). For instance, Zhu and colleagues have developed a hydrogel-based miRNAs delivery system to regenerate damaged cartilage by providing a regenerative milieu to limit chondrocyte senescence that predominantly leads to cartilage lesions in OA (Zhu et al., 2022). MiR-23a-3p is the most highly expressed in human umbilical cord mesenchymal stem cells-derived small extracellular vesicles. The latter demonstrates an effect in promoting cartilage regeneration by transferring miR-23a-3p (Hu et al., 2020). In a rat OA model, exosomes origin from miR-140-5p-overexpressing human synovial mesenchymal stem cells significantly promote cartilage regeneration and decelerate knee OA progression (Tao et al., 2017). Furthermore, Wang et al. have shown a strong cartilage regenerative capacity of the miR-221-3p-containing EVs, which are derived from chondrogenic progenitor cells of MRL/MpJ superhealer mice, in the destabilization of the medial meniscus (DMM)-induced OA model (Wang et al., 2020).

### Dysregulated miRNAs expression in OA

The miRNA profiles during the initiation and progression of OA have been characterized in human OA cartilage and two surgically-induced mouse OA models, including the DMM model and the anterior cruciate ligament transection (ALCT) model. By serum miRNAs microarray analysis, Ntoumou et al. identified 279 differentially expressed miRNAs in serum samples of OA patients as compared with healthy controls, among which miR-140-3p, miR-33b-3p, and miR-571-3p could be used as potential biomarkers of OA (Ntoumou et al., 2017). Almeida and colleagues performed RNA sequencing in human-preserved and lesioned OA cartilage to profile the changes in miRNA and mRNA (Coutinho de Almeida et al., 2019). Their data showed a total of 142 miRNAs and 2387 mRNA that were differentially expressed between preserved and damaged OA cartilage. By bioinformatic approaches, a miRNA-mRNA interactome network was drawn consisting of 62 miRNAs targeting 238 mRNA to elucidate the molecular regulatory network during OA pathogenesis (Coutinho de Almeida et al., 2019). In the DMM model, a total of 139 miRNAs were determined to be differentially expressed in OA articular cartilage at one and/or 6 weeks after surgery (Kung et al., 2018). A paired miRNA/mRNA expression analysis confirmed an overlapping dysregulation of miRNAs between mouse OA cartilage and human end-staged OA cartilage. The overlapped miRNAs include miR-15/16-5p, miR-26p-5p, miR-30c-5p, miR-98-5p, miR-149-5p, miR-210-3p and miR-342-3p. Moreover, some unreported OA-associated miRNAs were discovered, including miR-574-5p, miR-31-5p, and let-7d-5p. In a rat ALCT model, Cheng and colleagues analyzed the miRNA profile after ALCT surgery with or without extracorporeal shockwave (SW) therapy (Cheng et al., 2016). By the next-generation sequencing technology, 118 differentially expressed miRNAs were identified in articular cartilage, and 214 differentially expressed miRNAs were identified in the subchondral bone. Global functional analysis revealed that the identified miRNAs were associated with cartilage development, inflammatory response, cell adhesion, transcription and translation, cell cycle, signal transduction, apoptotic process, collagen fibril organization, and chondrocyte differentiation (Cheng et al., 2016). Moreover, Zheng et al. demonstrated a significantly higher level of serum miR-98 expression in OA patients than in healthy individuals, suggesting miR-98 as a potential diagnostic biomarker for OA (Zheng et al., 2018). In a rat model of OA, upregulated expression of miR-98 was also observed, and the knockdown of miR-98 had an inhibitory effect on cartilage deterioration and chondrocyte death (Wang et al., 2016b).

### MiRNAs are involved in OA inflammation

Low-grade inflammation is a key player in OA pathogenesis (Robinson et al., 2016). IL-1β is the most important pro-inflammatory cytokine during early OA onset, and IL-1β treatment has been used to stimulate chondrocyte apoptosis and ECM catabolism to mimic OA phenotype *in vitro* (Wojdasiewicz et al., 2014). Cumulating evidence has revealed the participation of miRNAs in IL-1β-stimulated OA-like phenotypes in articular chondrocytes. For instance, miR-377-3p alleviates the chondrocyte apoptosis under IL-1β stimulation *in vitro* and the damage of synovial tissues in facet joint OA *in vivo* (Tu et al., 2020). An association between increased miR-448 and decreased expression of matrilin-3 has been found in human OA cartilage tissues compared to normal cartilage (Yang et al., 2018). The deletion of miR-448 significantly ameliorates the IL-1β-induced catabolic phenotype in primary cultured chondrocytes, whereas overexpression of miR-448 shows the opposite effects (Yang et al., 2018). Martilin-3 is the target gene of miR-448, and the genetic deletion of matrilin-3 reverses the regulatory effects of miR-448 on the chondrocyte catabolism (Yang et al., 2018). The expression level of miR-320 is reduced in the IL-1β-treated primary mouse chondrocytes (PMCs) (Meng et al., 2016). Forced expression of miR-320 inhibits the expression of matrix proteinase Mmp13 by targeting its 3′-UTR of mRNA, whereas anti-miR-320 treatment enhances the Mmp13 expression (Meng et al., 2016). Activating the NFκB/MAPK pathway downregulates the miR-320 expression in the IL-1β-treated PMCs (Meng et al., 2016). MiR-320c inhibits the expression and transcriptional activity of β-catenin, whereas loss of miR-320c expression leads to OA-like phenotypes in chondrocytes and late-stage chondrogenic differentiation in human adipose-derived stem cells (Zhang et al., 2018a; Hu et al., 2019a). Injection of miR-320-3p agonist attenuates OA progression in the OA mouse model (Hu et al., 2019a). However, controversial results have also been reported. Peng et al. reported an increased miR-320a expression in OA chondrocytes as compared with normal chondrocytes. They further showed that transfection of miR-320 antagonists inhibits the Mmp13 expression in human OA chondrocytes *in vitro* by regulating the expressions of BMI-1 and Runx2 mRNA (Peng et al., 2017). The possible reasons for these controversial results could be the small sample size (N = 5–6) in these studies and the heterogeneity of primary human chondrocytes isolated from OA patients. IL-1β treatment downregulates the level of miR-27a-3p and increases the expression of its target gene ADAMTS5 mRNA in the primary human chondrocytes (Li et al., 2018). Overexpression of miR-27a-3p abolishes the expression of Adamts5 induced by the IL-1β treatment (Li et al., 2018). Furthermore, Qiu and colleagues have demonstrated that miR-27a suppresses the inflammatory response and catabolic activity during IL-1β stimulation in chondrocytes by targeting toll-like receptor 4 (TLR4) (Qiu et al., 2019). Transfection of miR-27a mimics significantly reduces the production of reactive oxygen species (ROS) and a series of pro-inflammatory cytokines, including IL-6, IL-8, and TNF-α in chondrocytes (Qiu et al., 2019).

TNF-α is another crucial inflammatory cytokine in OA pathogenesis (Robinson et al., 2016). Hu et al. reported a role of miR-145 in TNF-α-driven cartilage matrix degradation during the OA progression (Hu et al., 2017). MiR-145 expression is dramatically decreased in TNF-α-treated chondrocytes and in OA cartilages (Hu et al., 2017). MiR-145 could directly target the 3′UTR of mitogen-activated protein kinase 4 (MKK4) mRNA to inhibit the production of several TNF-α-induced matrix-degrading proteinases, including Mmp3, Mmp13, and Adamts5 (Hu et al., 2017). Intraarticular injection of miR-145 alleviates cartilage degradation and matrix catabolism in a rat DMM model (Hu et al., 2017).

Lipopolysaccharide (LPS) is one of the most crucial pro-inflammatory factors, stimulating strong inflammatory responses in synovial tissues and cartilage in arthritic diseases. In LPS-stimulated primary chondrocytes, Ding et al. have shown that overexpression of miR-93 significantly enhances chondrocyte viability and inhibits chondrocyte apoptosis (Ding et al., 2019). Transfection of miR-93 mimics in primary chondrocytes depresses the expression of several LPS-induced inflammatory cytokines, including TNF-α, IL-1β, and IL-6 both *in vitro* and *in vivo* (Ding et al., 2019). Another report from Zhang et al. has shown that miR-9 is significantly reduced in articular cartilage tissues from OA patients (Zhang et al., 2018b). In a sodium iodoacetate-induced rat OA model, the authors demonstrate that intraarticular injection of miR-9 agomir significantly downregulates the expression level of matrix proteinase Mmp13 and upregulates the expression level of Col2a1. Mechanistically, miR-9 directly binds to the 3′-UTR of the Mmp13 mRNA (Zhang et al., 2018b). Furthermore, miR-26a has also been reduced in rat OA models (Zhao et al., 2019a). MiR-26a plays a vital role in inhibiting the phosphorylation levels of IκBα and p65, two important factors in the NFκB inflammatory signaling pathway, thus ameliorating synovial hyperplasia and cartilage injury during OA progression (Zhao et al., 2019a). A low expression level of miR-107 has been found in chondrocytes from OA patients compared with normal controls (Zhao et al., 2019b). Transfection of miR-107 mimics into chondrocytes inhibits apoptosis and promotes autophagy by activating AKT/mTOR and NF-κB pathway (Zhao et al., 2019b).

### MiRNAs mediate epigenetic modifications in OA

Recently, changes in miRNAs have been linked with epigenetic regulations. In primary cultured human OA chondrocytes, Wu and coworkers have demonstrated that miR-200b-3p targets the expression of DNA methyltransferase 3 alpha (DNMT3A) to regulate the secretion of matrix proteinases and synthesis of type II collagen (Wu et al., 2017). In IL-1β-stimulated chondrocytes, Ma et al. have shown that miR-33b-3p also targets DNMT3A to inhibit IL-1β-induced chondrocyte apoptosis as well as ECM degradation (Ma et al., 2019). Chen and colleagues have reported that miR-455-3p regulates the expression of histone deacetylase 2/8 (HDAC2/8) (Chen et al., 2016). Their results further showed that miR-445-3p downregulates the expression of HDAC2/8 and promotes histone H3 acetylation at the COL2A1 promoter in human chondrogenic cells (Chen et al., 2016). Results from a different research group demonstrated that miR-455-3p targets P21-activated kinases 2(PKA2) to promote the TGF-β signaling pathway and inhibit OA progression (Hu et al., 2019b). MiR-95-5p promotes chondrogenesis and thus inhibits the progression of OA by targeting HDAC2/8 (Mao et al., 2018a). The same research group further reported a role of miR-92a-3p overexpressing exosomes from human mesenchymal stem cells in promoting chondrogenesis and preventing cartilage degradation through regulating histone deacetylase 2 and Wnt5a (Mao et al., 2018b).

### Exosomal miRNAs in OA pathogenesis

Exosomal miRNAs are identified to be pivotal mediators of intercellular communication and thus involved in OA pathogenesis (Fan et al., 2022). Interestingly, Liu and coworkers found a significant increase in circulating exosomal osteoclast-derived miRNAs during the early OA onset (Liu et al., 2021). Targeted deletion of osteoclast-derived miRNAs by genetic knockout of the essential miRNA-processing enzyme Dicer or blocking the release of osteoclast-derived exosomes by siRNA-mediated silencing of Rab27a markedly limited ECM degeneration, osteochondral angiogenesis, and sensory innervation in a surgery-induced mouse OA model (Liu et al., 2021). It has been further demonstrated that osteoclast-derived miRNAs caused OA-like lesions through suppressing the tissue inhibitor of metalloproteinase-2 (TIMP-2) and TIMP-3 (Liu et al., 2021). Furthermore, the authors utilized their previously established osteoclast-targeted delivery system to show that systemic inhibition of osteoclast exosome largely mitigates the surgery-induced OA lesions in mice (Liu et al., 2015; Liu et al., 2021). On the other hand, it has been reported that exosomes derived from mesenchymal stem cells contain certain clusters of miRNAs, such as miR-100-5p and miR-127-3p, which can protect against OA damages and ameliorate gait abnormalities (Tao et al., 2017; Wu et al., 2019; Qiu et al., 2020; Dong et al., 2021; Tao et al., 2021; Xu and Xu, 2021). The above studies have clearly shown that exosomal miRNAs play a central role in the pathogenesis of OA; nonetheless, the complex networks of these exosomal miRNAs still warrant further investigations.

### MiRNAs as prognostic indicators for OA

Recent studies have highlighted miRNAs as the potential indicators of therapeutic effects for OA-treating drugs. In an *ex-vivo* porcine cartilage impact injury model, Genemaras et al. have tested whether interleukin-1 receptor antagonist protein (IRAP) inhibits impact injury-induced inflammation and catabolism in chondrocytes (Genemaras et al., 2015). The data showed that IRAP significantly decreases the expression levels of matrix-degrading enzymes (*i.e.,* Adamts4/5 and Mmp13) as well as inflammatory cytokines (*i.e.,* IL-1β and TNF-α) *via* downregulation of a series of miRNAs, including miR-140, miR-125b and miR-27b (Genemaras et al., 2015). In 2018, the same research group further reported the effects of anti-inflammatory agents, including IRAP, hyaluronan, dexamethasone, and mesenchymal stem cell treatment on genetic markers of miRNAs, cartilage matrix degradation, apoptosis, and inflammation in an *ex-vivo* porcine model of acute knee cartilage injury (Genemaras et al., 2018). IRAP significantly increases the expression of miR-140, miR-125b, miR-27b, miR-146a, and miR-22 in injured cartilage. Hyaluronan treatment increases the expression of miR-34a in addition to the above miRNAs. MiR-140 has been linked to chondrogenesis and cartilage formation (Miyaki et al., 2010). Loss of miR-140 in mice causes dysplasia due to impaired chondrocyte proliferation. Results from two groups have demonstrated that estrogen treatment inhibits cartilage degradation and the secretion of Mmp13 from chondrocytes by regulating the expression level of miR-140 (Liang et al., 2016; Xu et al., 2019). The expression level of miR-140 was positively correlated with the therapeutic effects of estrogen in an ovariectomized (OVX) rat model of postmenopausal OA. Estrogen treatment significantly increases the miR-140-5p level compared with the control group, whereas knockdown of miR-140 expression abolishes the inhibitory effect of estrogen on cartilage degradation. Moreover, miR-140 expression can be upregulated by melatonin treatment, leading to enhanced cell proliferation, promoted expression of cartilage ECM proteins (e.g., type II collagen and aggrecan), and inhibited levels of proteinases, including MMP9/13 and ADAMTS4/5, in IL-1β-treated human chondrocytes (Zhang et al., 2019). Taken together, the above findings suggest that miRNAs play central roles in the pathological mechanisms of OA and could serve as diagnostic and prognostic biomarkers and potential therapeutic targets for OA treatment. The functions of miRNAs in cartilage homeostasis and OA pathogenesis are summarized in Table 1.

**TABLE 1:** Roles of miRNAs in cartilage homeostasis and OA pathogenesis.

MiRNAs	Targets	Functions	References
miR-204/-211	Runx2	It maintains cartilage homeostasis to protect against OA initiation	Huang et al. (2019)
miR-193b-3p	HDAC3	It regulates hMSC chondrogenesis and metabolism	Meng et al. (2018)
miR-200a	Col1a1, Sox9	It controls chondrocyte proliferation and differentiation	Umeda et al. (2015)
miR-9	protogenin, Mmp13	It regulates chondrogenesis and ECM metabolism	(Song et al., 2013) (Zhang et al., 2018b)
miR-142-3p	HMGB1	It inhibits chondrocyte apoptosis and inflammation	Wang et al. (2016a)
miR-140	Adamts5, Mmp13	It regulates chondrocyte proliferation, ECM composition, and cartilage regeneration	(Miyaki et al., 2010; Si et al., 2017; Tao et al., 2017; Zhang et al., 2019)
miR-221/-222	CDKN1B/p27, TIMP-3, Tcf7l2/TCF4, ARNT	They are involved in mechanotransduction, gene expression, and cartilage regeneration	(Dunn et al., 2009; Wang et al., 2020; Shang et al., 2021)
miR-455	Hif2-α	It protects cartilage from OA-like damages	Ito et al. (2021)
miR-17	Hif1-α	It regulates the ECM metabolism in cartilage	Zhang et al. (2022)
miR-98	Bcl-2	It prevents chondrocyte apoptosis under IL-1β stimulation and can serve as a potential diagnostic biomarker for OA.	(Wang et al., 2016b; Zheng et al., 2018)
miR-377-3p	Itga6	It attenuates IL-1β-induced chondrocyte apoptosis and catabolism	Tu et al. (2020)
miR-448	Martilin-3	It promotes IL-1β-induced chondrocyte catabolism	Yang et al. (2018)
miR-320	Mmp13, β-catenin, BMI-1, Runx2	It regulates chondrocyte differentiation and ECM metabolism	(Meng et al., 2016; Peng et al., 2017; Zhang et al., 2018a; Hu et al., 2019a)
miR-27a	Adamts5, Tlr4	It regulates inflammatory response during IL-1β stimulation	(Li et al., 2018; Qiu et al., 2019)
miR-145	Mmk4	It directly targets 3′-UTR of Mmk4 mRNA.	Hu et al. (2017)
miR-93	Tlr4	It attenuates LPS-induced chondrocyte apoptosis and inflammation	Ding et al. (2019)
miR-26a	IκBα, p65	It inhibits NF-κB pathway to reduce synovial inflammation and cartilage injury	Zhao et al. (2019a)
miR-107	Traf3	It regulates chondrocyte autophagy and apoptosis	Zhao et al. (2019b)
miR-128a	Atg12	It suppresses the autophagy of chondrocytes and thus aggravates OA development	Lian et al. (2018)
miR-200b	Dnmt3	It promotes ECM anabolism and proliferation of OA chondrocytes	Wu et al. (2017)
miR-33b-3p	Dnmt3	It inhibits IL-1β-induced chondrocyte apoptosis and ECM degradation	Ma et al. (2019)
miR-445-3p	HDAC2/8, Pak2	It promotes Col2al expression and enhances TGF-β signalling to prevent OA progression	(Chen et al., 2016; Hu et al., 2019b)
miR-95-5p	HDAC2/8	It regulates chondrogenesis and ECM metabolism	Mao et al. (2018a)
miR-92a-3p	HDAC2, Wnt5a	It regulates chondrogenesis and ECM metabolism	Mao et al. (2018b)
miR-29b-5p	TET1	It promotes cartilage regeneration by suppressing chondrocyte senescence	Zhu et al. (2022)
miR-23a-3p	PTEN	It promotes cartilage regeneration	Hu et al. (2020)
osteoclast-derived exosomal miRNAs	TIMP-2, TIMP-3	It can be transferred to articular chondrocytes to promote ECM degeneration, osteochondral angiogenesis, and sensory innervation	Liu et al. (2021)
MSCs-derived exosomal miRNAs	GIT1, NFκB, WNT5A	It can be transferred to chondrocytes to protect against OA damages and ameliorate gait abnormalities	(Tao et al., 2017; Wu et al., 2019; Qiu et al., 2020; Dong et al., 2021; Tao et al., 2021; Xu and Xu, 2021)

### LncRNAs maintain cartilage homeostasis

Under physiological conditions, lncRNA expression is pivotal for regulating gene expression and maintaining cartilage homeostasis. LncRNAs can regulate chondrocyte differentiation by mediating the expression of Sox9, BMP7, and IGF-2 (Zhu et al., 2019). Interestingly, a recent study demonstrated that lncRNA MM2P, stimulated by IL-4 or IL-13, induces expression and exosomal transfer of Sox9 mRNA from monocyte-derived cells to primary chondrocytes to maintain a healthy phenotype of chondrocytes (Bai et al., 2020). The expressions of LncRNA KLF3-AS1 (KLF3 antisense RNA 1) and metastasis-associated lung adenocarcinoma transcript 1 (MALAT-1) are essential for the regulatory effects of hMSCs-derived exosomes on ECM metabolism, chondrocyte differentiation, inflammation, to maintain cartilage homeostasis (Liu et al., 2018; Pan et al., 2021). Some studies have reported that lncRNAs promote chondrocyte proliferation while inhibiting the chondrocyte apoptosis (Jiang et al., 2019; Wang et al., 2021; Xie et al., 2021). For instance, lncRNA small nucleolar RNA host gene 5 (SNHG5) can act as a sponger of miR-10a-5p to suppress chondrocytes apoptosis (Jiang et al., 2021). Loss of SNHG5 expression leads to enhanced IL-1β-induced chondrocyte apoptosis. SNHG15 maintains ECM homeostasis to protect against OA damages (Chen et al., 2020a). Zhang et al. also confirmed the protective role of SNHG15 during OA initiation and progression as SNHG15 can competitively bind with miR-141-3p to upregulate the expression of BCL2L13 (Zhang et al., 2020a). LncRNA maternally expressed 3 (MEG3) promotes chondrocyte proliferation and migration and inhibits apoptosis and inflammation (Huang et al., 2021). Collectively, current evidence has suggested that lncRNA expression is essential for the maintenance of cartilage homeostasis.

### LncRNAs mediate cartilage regeneration

Several studies have recently shown that lncRNAs play an essential role in cartilage regeneration. Wang et al. reported that lncRNA colorectal neoplasia differentially expressed gene (CRNDE) regulates the levels of silent information regulator factor 2-related enzyme 1 (SIRT1) and Sox9 to promote the chondrogenic differentiation of bone marrow mesenchymal stem cells (BMSCs) *in vitro* and enhance cartilage regeneration in a rat model of OA (Shi et al., 2021). LncRNA KLF3-AS1 is markedly enriched in hMSCs-derived exosomes, and the latter is demonstrated to promote cartilage repair *via* enhancing chondrocyte proliferation in a rat model of OA (Pan et al., 2021). LncRNA H19 can transfer from umbilical cord mesenchymal stem cells (UMSCs) to chondrocytes *via* exosomes and act as a competing endogenous sponge of miR-29b-3p and miR-29b-3p to regulate FOXO3 expression (Yan et al., 2021). Intra-articular injection of H19-containing exosomes facilitates cartilage repair in an *in vivo* SD rat cartilage defect model (Yan et al., 2021). Moreover, exosomal H19 derived from UMSCs and fibroblast-like synoviocytes-derived promotes chondrocyte migration and ECM synthesis while suppressing chondrocyte senescence and apoptosis, both *in vitro* and *in vivo* (Tan et al., 2020; Yan et al., 2021).

### Dysregulated LncRNAs expression in OA

Recent studies have suggested lncRNAs as promising candidates for the diagnosis and therapy of OA as their expression is significantly changed during OA initiation and progression (Okuyan and Begen, 2022). The lncRNA profile under physiological and OA conditions has been analyzed by Hoolwerff and colleagues (van Hoolwerff et al., 2020). LncRNA sequencing was conducted in preserved and lesioned OA articular cartilage tissues from patients taking total knee replacement. The data showed a difference of 191 lncRNAs between preserved and lesioned OA cartilage. The identified lncRNAs were classified into trans-acting lncRNA or cis-acting lncRNA based on their functional location relative to the transcription site. By comparing the distribution of dysregulation of lncRNAs with all transcriptional mRNAs and all sense genes with differentially expressed antisense lncRNAs, the data suggested a cis-regulation mechanism for both intergenic and antisense lncRNAs in OA cartilage. To validate the above cis-regulation mechanism of lncRNAs, the authors chose P3H2-AS1 as an example for proof of concept. A P3H2-AS1 targeting locked nucleic acid (LNA) GapmeRs was transfected into primary chondrocytes, and the expression level of P3H2 was determined. The results showed a significant decrease in P3H2 expression in cells transfected with targeting LNA GapmeRs compared to those transfected with non-targeting LNA GapmeRs. Chen and colleagues Field also analyzed the dysregulation of lncRNAs and related mRNA network (Chen and Chen, 2020). They detected a total of 49 lncRNAs and 1212 mRNAs that were differentially expressed in OA knee articular cartilage as compared with normal controls. In this lncRNA-mRNA network, 7 hub lncRNAs were identified, including MIR210HG, LINC00313, LINC00839, TBC1D3P1-DHX40P1, ISM1-AS1, LINC00654, and HCP5. Further Kyoto encyclopedia of genes and genomes (KEGG) analysis revealed that these OA-related hub lncRNAs were associated with osteoclast differentiation, the FoxO signaling pathway, the TNF signaling pathway, the P53 signaling pathway, and extracellular matrix organization.

### LncRNAs mediate inflammation in OA

LncRNA ADP-ribosylation factor-related protein 1 (ARFRP1) was found to be increased in OA cartilage and in the LPS-treated chondrocytes (Zhang et al., 2020b). Loss of ARFRP1 ameliorated LPS-induced chondrocyte injury *via* regulating miR-15a-5p/TLR axis. Growth arrest specificity 5 (GAS5) was found to be upregulated in serum and cartilage tissues from knee OA patients (Gao et al., 2020). Overexpression of GAS5 induced chondrocyte apoptosis and inhibited chondrocyte proliferation through downregulation of miR-137. The expression of MALAT1 was reported to be reduced in OA patients (Li et al., 2020). Loss of MALAT1 promotes the production of cyclooxygenase-2 (COX-2), IL-6, and Mmp13 and inhibits the level of type II collagen in the LPS-treated chondrocytes (Li et al., 2020). MALAT1 regulates miR-146a to control PI3K/Akt/mTOR pathway during LPS-induced chondrocyte catabolism, inflammation, and apoptosis (Li et al., 2020). However, another report from Liu et al. showed a controversial role of MALAT1 in the OA pathogenesis (Liu et al., 2019). Their data showed upregulation of MALAT1 in OA cartilages and IL-1β-stimulated chondrocytes. Overexpression of MALAT1 depressed chondrocyte viability and enhanced cartilage catabolism *via* upregulation of Adamts5 during IL-1β treatment. The nuclear enriched abundant transcript 1 (NEAT1), the prostate cancer-associated transcript 1 (PCAT1), and the SNHG5 can respectively regulate chondrocyte proliferation and apoptosis *via* controlling several downstream miRNAs (Jiang et al., 2021; Xiao et al., 2021; Zhou et al., 2021). An association between decreased expression of OIP5 antisense RNA 1 (OIP5-ASI) and increased expression of miR-29b-3p was found in IL-1β-treated CHON-001 and ATDC5 chondrocyte-like cells (Zhi et al., 2021). Overexpression of OIP5-ASI increases chondrocyte viability and proliferation and decreases the production of inflammatory cytokines. OIP5-ASI directly binds to miR-29-3p to control the expression of PGRN.

### LncRNAs mediate ECM metabolism in OA

The plasmacytoma variant translocation 1 (PVT1) and the HOXA transcript at the distal tip (HOTTIP) can participate in the ECM degradation during OA progression (Mao et al., 2019; Lu et al., 2020). PVT1 expression is elevated in OA patients and IL-1β-stimulated C28/I2 chondrocytes (Lu et al., 2020). Deletion of PVT1 enhanced cell survival and autophagy and depressed IL-1β-induced apoptosis and inflammation. PVT1 loss upregulated the levels of miR-27b-3p and downregulated downstream target mRNAs of miR-27b-3p. Another study suggests that PVT1 is involved in the hyperglycemia-induced collagen degradation, probably through regulation of the miR-26b-TGF-β1-axis (Ding et al., 2020). LncRNA LINC00671 exacerbates OA lesions by enhancing ONECUT2-mediated Smurf2 expression and ECM degradation (Chen and Xu, 2021).

### LncRNAs mediate epigenetic modifications in OA

HOX antisense intergenic RNA (HOTAIR) is found to be elevated in OA chondrocytes (Yang et al., 2020). Loss- and gain-of-function studies demonstrated that HOTAIR directly targets Wnt inhibitory factor 1 (WIF-1) by promoting the H3K27 trimethylation (Yang et al., 2020). Overexpression of HOTAIR increased expression of BMP2, Mmp13, and Adamts5 and decreased expression of Sox9 in SW1353 chondrocyte-like cells, and silencing of HOTAIR exerted opposite effects (Yang et al., 2020). HOTAIR acts as a sponge to regulate the expression of miR-130a-3p and miR-20b to control chondrocyte autophagy and catabolism (Chen et al., 2020b; He and Jiang, 2020; Yang et al., 2020). LncRNA X inactive specific transcript (XIST) contributes to OA progression *via* miR-149-5p/DNMT3A axis (Liu et al., 2020). Lysyl oxidase-like 1 antisense RNA 1 (LOXL1-AS1) can sponge miR-423-5p and abolish miR-423-5p-dependent inhibition on lysine demethylase 5C (KDM5C) to promote OA progression (Chen et al., 2020c). Taken together, these findings suggest lncRNAs as potential therapeutic targets for OA treatment. The roles of lncRNAs in cartilage homeostasis and OA pathogenesis are summarized in Table 2.

**TABLE 2:** Role of lncRNAs in cartilage homeostasis and OA pathogenesis.

LncRNAs	Targets	Functions	References
MM2P	Stat3	It induces the expression and exosomal transfer of Sox9 mRNA *via* activating Stat3	Bai et al. (2020)
KLF3-AS1	miR-206	It enhances chondrocyte proliferation to promote cartilage repair	Liu et al. (2018)
THUMPD3-AS1	?	It enhances chondrocyte proliferation and inflammatory response	Wang et al. (2021)
MEG8	PI3K/AKT signaling	It regulates chondrocyte cell proliferation, apoptosis, and inflammation	Xie et al. (2021)
PACER	HOTAIR	It regulates chondrocyte apoptosis and lncRNA HOTAIR expression	Jiang et al. (2019)
SNHG5	miR-10a-5p	It regulates chondrocyte proliferation and apoptosis	Jiang et al. (2021)
SNHG15	KLF4, miR-7, miR-141-3p	It regulates ECM metabolism in OA.	(Chen et al., 2020a; Zhang et al., 2020a)
MEG3	miR-9-5p	It protects chondrocytes from IL-1β-induced inflammation	Huang et al. (2021)
ARFRP1	miR-15a-5p	It promotes LPS-induced cartilage injury	Zhang et al. (2020b)
GAS5	miR-137	It induces chondrocyte apoptosis	Gao et al. (2020)
LOXL1-AS1	miR-423-5p	It controls KDM5C expression to promote OA.	Chen et al. (2020c)
MALAT1	miR-146a, miR-145	It regulates the mTOR signaling pathway, chondrocyte viability, and ECM degradation	(Liu et al., 2019; Li et al., 2020)
NEAT1	miR-543	It regulates chondrocyte proliferation and apoptosis	Xiao et al. (2021)
PCAT-1	miR-27-3p	It regulates chondrocyte apoptosis	Zhou et al. (2021)
PVT1	miR-27b-3p, miR-26b	It regulates the expression of TRAF3 and CTGF/TGF-β pathway	(Ding et al., 2020; Lu et al., 2020)
HOTTIP	miR-455-4p	It promotes CCL3 expression *via* sponging miR-455-3p	Mao et al. (2019)
XIST	miR-149-5p	It promotes OA progression *via* regulation of miR-149-5p/DNMT3A axis	Liu et al. (2020)
LINC00671	ONECUT2	It exacerbates OA lesions by regulating Smurf2 expression	Chen and Xu, (2021)
OIP5-ASI	miR-29b-3p	It inhibits IL-1β-induced chondrocyte apoptosis and inflammatory response	Zhi et al. (2021)
HOTAIR	WIF-1, miR-130a-3p, miR-20b	It regulates WIF-1 and PTEN expression and chondrocyte autophagy	(Chen et al., 2020b; He and Jiang, 2020; Yang et al., 2020)
CRNDE	SIRT1, SOX9	It promotes cartilage repair through enhancing BMSC chondrogenic differentiation	Shi et al. (2021)
H19	miR-29b-3p, miR-106b-5p	It alleviates OA progression and improves osteochondral activity	(Tan et al., 2020; Yan et al., 2021)

## Conclusion and perspectives

**FIGURE 1:**
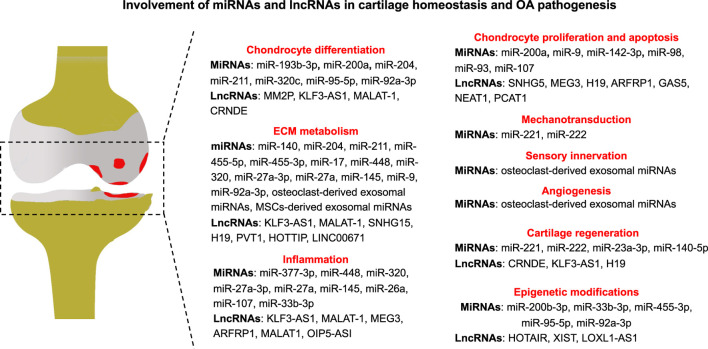
Involvement of miRNAs and lncRNAs in cartilage homeostasis and OA pathogenesis.

In this review, we discussed the functional roles of ncRNAs, especially miRNAs and lncRNAs, in cartilage homeostasis and OA pathogenesis (Figure 1). RNA sequencing data reveal a bunch of ncRNAs associated with OA initiation and progression. Nonetheless, the mechanism underlying how ncRNAs regulate cartilage homeostasis and participate in OA pathogenesis remains elusive. Several loss- and gain-of-function studies utilizing transgenic animal models have suggested that ncRNAs exert vital functions in maintaining cartilage homeostasis, and manipulations on ncRNA expression can promote or decelerate the progression of OA through direct or indirect molecular mechanisms. Moreover, ncRNAs can serve as promising diagnostic biomarkers, prognostic indicators, and therapeutic targets for OA. However, we must point out that the quality of current evidence regarding ncRNAs and their functions in OA is relatively low due to inappropriate study design, controversial results, and the lack of direct *in vivo* evidence. Further high-quality investigations are still needed to confirm and characterize the functional role of ncRNAs in OA pathogenesis in the future.
